# Prognosis and Risk Factors for Congenital Airway Anomalies in Children with Congenital Heart Disease: A Nationwide Population-Based Study in Taiwan

**DOI:** 10.1371/journal.pone.0137437

**Published:** 2015-09-03

**Authors:** Yu-Sheng Lee, Mei-Jy Jeng, Pei-Chen Tsao, Wen-Jue Soong, Pesus Chou

**Affiliations:** 1 Division of General Pediatrics, Department of Pediatrics, Taipei Veterans General Hospital, Taipei, Taiwan; 2 Department of Pediatrics, National Yang-Ming University School of Medicine, Taipei, Taiwan; 3 Institute of Public Health and Community Medicine Research Center, National Yang-Ming University School of Medicine, Taipei, Taiwan; 4 Institute of Emergency and Critical Care Medicine, National Yang-Ming University School of Medicine, Taipei, Taiwan; University of Southern California, UNITED STATES

## Abstract

**Background:**

The mortality risk associated with congenital airway anomalies (CAA) in children with congenital heart disease (CHD) is unclear. This study aimed to investigate the factors associated with CAA, and the associated mortality risk, among children with CHD.

**Methods:**

This nationwide, population-based study evaluated 39,652 children with CHD aged 0–5 years between 2000 and 2011, using the Taiwan National Health Insurance Research Database (NHIRD). We performed descriptive, logistic regression, Kaplan–Meier, and Cox regression analyses of the data.

**Results:**

Among the children with CHD, 1,591 (4.0%) had concomitant CAA. Children with CHD had an increased likelihood of CAA if they were boys (odds ratio [OR], 1.48; 95% confidence interval [CI], 1.33–1.64), infants (OR, 5.42; 95%CI, 4.06–7.24), or had a congenital musculoskeletal anomaly (OR, 3.19; 95%CI, 2.67–3.81), and were typically identified 0–3 years after CHD diagnosis (OR, 1.33; 95%CI 1.17–1.51). The mortality risk was increased in children with CHD and CAA (crude hazard ratio [HR], 2.05; 95%CI, 1.77–2.37), even after adjusting for confounders (adjusted HR, 1.76; 95%CI, 1.51–2.04). Mortality risk also changed by age and sex (adjusted HR and 95%CI are quoted): neonates, infants, and toddlers and preschool children, 1.67 (1.40–2.00), 1.93 (1.47–2.55), and 4.77 (1.39–16.44), respectively; and boys and girls, 1.62 (1.32–1.98) and 2.01 (1.61–2.50), respectively.

**Conclusion:**

The mortality risk is significantly increased among children with CHD and comorbid CAA. Clinicians should actively seek CAA during the follow-up of children with CHD.

## Introduction

Congenital heart disease (CHD) is a gross structural anomaly of the heart or intrathoracic great vessels that is present at birth and manifests with actual or potential functional significance [1]. It is one of the most common major congenital anomalies, with a reported prevalence at birth that varies worldwide from 5 to 8 per 1,000 live births [2–4]. Because of progress made in neonatology, intensive care, pediatric cardiology, and cardiovascular surgery, CHD is no longer considered a fatal disease, and can have favorable outcomes in many cases [5]. Therefore, it is crucial to explore the comorbid conditions and risk factors associated with CHD morbidity and mortality to improve the quality of life among these patients.

Aside from cardiac complications, non-cardiac comorbidities may also influence the health of patients with CHD [6, 7]. It is known that there is a higher prevalence of extracardiac anomalies (ECA) that can markedly affect morbidity and mortality among patients with CHD [8–13]. Congenital airway anomalies (CAA) are one such ECA believed to be significantly associated with CHD [5, 14–17].

Most studies to date have focused on the association of all ECA categories with CHD [8–13]. Concerning CAA in CHD, previous efforts were only concerned with the features of specific respiratory anomaly associated with specific cardiovascular anomaly or surgery [5, 14–19]. With respect to the associations between CAA and CHD, the available epidemiological information is scant, producing disparate results [5, 14–16], and no large-scale population-based studies have examined the correlation in detail. Furthermore, previous studies have not investigated the overall or age- and sex-stratified mortality risk among children with CHD and CAA.

The National Health Insurance Research Database (NHIRD) in Taiwan offers a nationwide population-based, multi-institutional database for research purposes. The aim of this study was to use the NHIRD to examine the associations between CAA and CHD, and to evaluate the mortality risk associated with CAA among children with CHD.

## Materials and Methods

### Ethics Statement

The Institutional Review Board of Taipei Veterans General Hospital, Taiwan, approved this study. Because all personal identifying information had been encrypted before the database was released, the review board requirement for written informed consent was waived.

### Data Sources

This study was based on data from the NHIRD released by the National Health Research Institute (NHRI). Taiwan began the National Health Insurance (NHI) program in 1995 to provide comprehensive health care for its population. Enrollment in the NHI program is mandatory and data presently exists for more than 23 million people, representing approximately 99% of Taiwan’s population [20]. The NHI program offers integrated medical care, including outpatient, inpatient, emergency, dental, and traditional Chinese medicine services, as well as medicine prescriptions. The NHIRD is managed and publicly released by the NHRI, and contains data for the reimbursement claims for medical care, ranging from demographic data to detailed orders. These features make the NHIRD one of the largest and most complete national health care service datasets in the world. The diagnostic codes of the patients in the NHIRD are in the format of the International Classification of Diseases, Ninth Revision, Clinical Modification (ICD-9-CM), and are established by board-certified physicians in their corresponding specialties. The diagnostic accuracy of the major diseases in the NHIRD has been validated [21–23].

All information that may potentially identify an individual patient was encrypted before the release of the database. The confidentiality of the NHIRD was maintained in accordance with the data regulations of the NHI Administration, Ministry of Health and Welfare and the NHRI, Taiwan. The NHRI guard the privacy of all beneficiaries and provide the NHIRD to researchers who have obtained ethical approval.

### Identification of the CHD Cohort

A retrospective population-based cohort study was conducted from January 1, 2000 to December 31, 2011. All inpatient data from NHIRD were collected for analysis over this 12-year period. Using the CHD diagnostic codes in the NHIRD (ICD-9-CM 745, 746, 747.0–4), 43,770 children with CHD, aged 0–5 years, were identified. Patients born before January 1, 2000 were excluded (n = 4,118). The final CHD study cohort comprised 39,652 children. The first date for each patient’s CHD diagnosis recorded in the NHIRD was defined as the index date. Information regarding the sex, age at CHD diagnosis, follow-up period, comorbidities, and in-hospital mortality was collected for analysis. The age at CHD diagnosis was grouped as follows: neonates (newborn up to 28 days), infants (aged more than 4 weeks but less than 12 months), and toddlers and preschool children (aged between 1 and 5 years). Mortality was defined according to the discharge status recorded in the NHIRD. All of the enrolled patients were followed-up until death or December 31, 2011, whichever was earlier.

### Identification of CAA and Other ECA

Patients with the diagnostic code for CAA, defined as 748 in the ICD-9-CM format [24], were identified from the database. Other associated congenital ECA were also recognized during the follow-up period. Based on the reports of previous studies [8, 9, 12, 13], we collected data for ECA of the nervous system (ICD-9-CM 740–742), eyes/ears/face/neck (ICD-9-CM 743–744), cleft lip and/or palate (ICD-9-CM 749), gastrointestinal system (ICD-9-CM 750.3,751), genitalia (ICD-9-CM 752), urinary system (ICD-9-CM 753), musculoskeletal system (ICD-9-CM 754–756), integumentary system (ICD-9-CM 757), and chromosomal anomalies (ICD-9-CM 758).

### Statistical Analysis

All data were linked by SQL server 2008 (Microsoft Corporation, Redmond, Washington, USA) and analyzed by the IBM SPSS statistics for Windows, Version 19.0 (IBM Corp., Armonk, NY, USA). The data were expressed as median (inter-quartile range, IQR) or percentage where appropriate. Chi-square or Fisher’s exact tests were used to assess the differences between the categorical variables. Multivariate logistic regression was applied to assess the associations between CAA and CHD. Odds ratios (OR) and 95%confidence intervals (CIs) are quoted as appropriate. The Kaplan–Meier analysis and log-rank test were used to estimate the cumulative incidence of mortality among children with CHD with and without CAA. Finally, Cox regression analyses with hazard ratios (HRs) were used to evaluate the mortality risk associated with CAA among children with CHD. For all tests, 2-tailed *p*-values < 0.05 were considered statistically significant.

## Results

### Demographic and Clinical Characteristics

During the 12-year study period, 39,652 children with CHD were enrolled, including 19,713 (49.7%) boys and 19,939 (50.3%) girls. Most of those with CHD were diagnosed as neonates (n = 22,147; 55.9%). The median follow-up period was 7 (3–9) years. The most common ECA among children with CHD was CAA (n = 1,591; 4.0%), followed by musculoskeletal (n = 1,250; 3.2%), and chromosomal (n = 1,267; 3.2%) anomalies. In total, 2,533 patients (6.4%) died during the follow-up, of which 199 (7.9%) had CAA.

CAA occurred in children with CHD at rate of 4.8% in boys and 3.2% in girls (*p* < 0.001). The CHD diagnostic age as infants (5.8%) and those in the first 0–3 years of follow-up had the highest rates of CAA in each sex and age stratum (*p* < 0.001). Children with all other ECA, except those with integumentary anomalies, had significantly higher rates of associated CAA (*p* < 0.001) (Table 1).

**Table 1:** Demographic and clinical characteristics among children with CHD.

	Total		CAA (+)		
Variables	n	(%)	n	(Rate, %)	*p* ^b^
No. of CHD patients	39652		1591	(4.0)	
Gender, n (%)					<0.001
Male	19713	(49.7)	946	(4.8)	
Female	19939	(50.3)	645	(3.2)	
Age at CHD diagnosis^a^, n (%)					<0.001
Neonates	22147	(55.9)	800	(3.6)	
Infants	12797	(32.3)	741	(5.8)	
Toddlers & Preschools	4708	(11.9)	50	(1.1)	
Median follow-up, years (IQR)	7	(3–9)			
Follow-up period, years					<0.001
0–3	10624	(26.8)	480	(4.5)	
4–7	11223	(28.3)	478	(4.3)	
≧8	17805	(44.9)	633	(3.6)	
Other associated ECA, n (%)					
Nervous system					<0.001
(+)	1183	(3.0)	157	(13.3)	
(-)	38469		1434	(3.7)	
Musculoskeletal system					<0.001
(+)	1250	(3.2)	202	(16.2)	
(-)	38402		1389	(3.6)	
Eyes/ears/face/neck					
(+)	475	(1.2)	85	(17.9)	<0.001
(-)	39177		1506	(3.8)	
Cleft lip/palate					<0.001
(+)	732	(1.8)	110	(15.0)	
(-)	8920		1481	(3.8)	
Gastrointestinal system					<0.001
(+)	846	(2.1)	81	(9.6)	
(-)	38006		1510	(3.9)	
Urinary system					<0.001
(+)	540	(1.4)	43	(8.0)	
(-)	39112		1548	(4.0)	
Genital system					<0.001
(+)	742	(1.9)	86	(11.6)	
(-)	38910		1505	(3.9)	
Integument					0.287
(+)	56	(0.1)	4	(7.1)	
(-)	39596		1587	(4.0)	
Chromosome					<0.001
(+)	1267	(3.2)	155	(12.2)	
(-)	38385		1436	(3.7)	
Mortality					<0.001
(+)	2533	(6.4)	199	(7.9)	
(-)	37119		1392	(3.8)	

Abbreviation: CHD, congenital heart disease; CAA, congenital airway anomaly; ECA, extracardiac anomaly; IQR, inter-quartile range.

^a^Neonate, newborn to ≤ 28 days of life; infant, >28 days but < 12 months; toddler & preschool, aged 1–5 years.

^b^χ^2^ or Fisher’s exact tests for the presence of CAA among different stratum

### Factors Associated with CAA in Children with CHD

Children with CHD had a significantly higher probability of having CAA if they were boys (OR, 1.43; 95%CI, 1.29–1.59), neonates (OR, 3.25; 95%CI, 2.43–4.35), or infants (OR, 5.31; 95%CI, 3.97–7.09) at CHD diagnosis. There was an elevated risk of diagnosis of CAA during the 0–3 years (OR, 1.33; 95%CI, 1.17–1.51) and 4–7 years (OR, 1.26; 95%CI, 1.11–1.43) after CHD diagnosis. Furthermore, there was a significantly greater probability of children with CHD having a CAA if they had any of the following ECA: musculoskeletal (OR, 3.19; 95%CI, 2.67–3.81), eyes/ears/face/neck (OR, 2.72; 95%CI, 2.07–3.56), nervous system (OR, 2.71; 95%CI, 2.25–3.28), chromosomal (OR, 2.51; 95%CI, 2.08–3.04), cleft lip/palate (OR, 2.28; 95%CI, 1.81–2.89), genital (OR, 1.76; 95%CI, 1.36–2.26), and gastrointestinal (OR, 1.59; 95%CI, 1.22–2.08) (Table 2).

**Table 2:** Multivariate logistic regression analysis for the presence of CAA among children with CHD.

Variables	aOR	(95%CI)^b^
Gender		
Male	1.43	(1.29–1.59)
Female	1.00	
Age at CHD diagnosis^a^		
Neonates	3.25	(2.43–4.35)
Infants	5.31	(3.97–7.09)
Toddlers & Preschools	1.00	
Follow-up period, years		
0–3	1.33	(1.17–1.51)
4–7	1.26	(1.11–1.43)
≧8	1.00	
Other associated ECA		
Nervous system		
(+)	2.71	(2.25–3.28)
(-)	1.00	
Musculoskeletal system		
(+)	3.19	(2.67–3.81)
(-)	1.00	
Eyes/ears/face/neck		
(+)	2.72	(2.07–3.56)
(-)	1.00	
Cleft lip/palate		
(+)	2.28	(1.81–2.89)
(-)	1.00	
Gastrointestinal system		
(+)	1.59	(1.22–2.08)
(-)	1.00	
Urinary system		
(+)	1.06	(0.74–1.52)
(-)	1.00	
Genital system		
(+)	1.76	(1.36–2.26)
(-)	1.00	
Integument		
(+)	0.94	(0.31–2.87)
(-)	1.00	
Chromosome		
(+)	2.51	(2.08–3.04)
(-)	1.00	

Abbreviations: CHD, congenital heart disease; CAA, congenital airway anomaly; ECA, extracardiac anomaly; aOR, adjusted odds ratio; CI, confidence interval.

Odds ratios (OR) and 95% confidence intervals (CIs) are quoted.

^a^Neonate, newborn to ≤ 28 days of life; infant, >28 days but < 12 months; toddler & preschool, aged 1–5 years.

^b^aOR adjusted for gender, age at CHD diagnosis, follow-up period and other ECA.

### Mortality Risk Associated with CAA in Children with CHD

**Fig 1:**
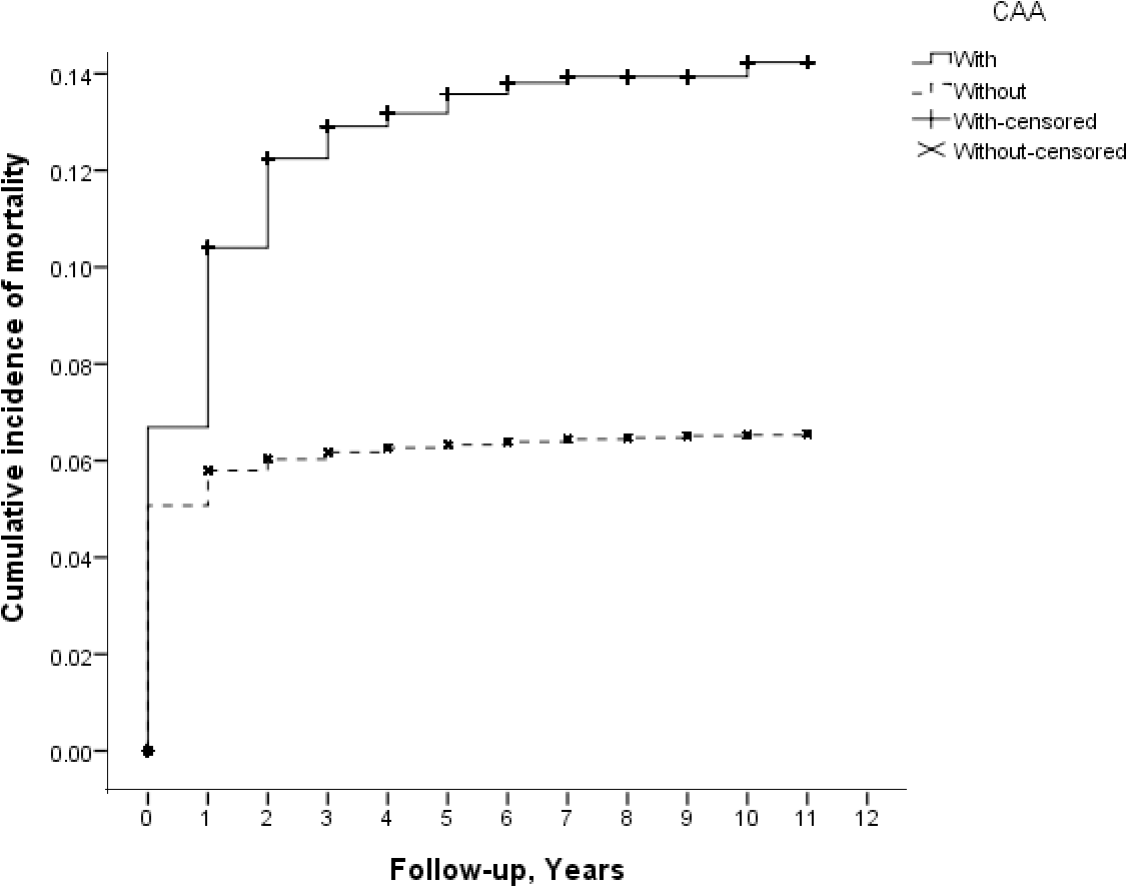
Cumulative incidence of mortality among CHD children with and without CAA, p < 0.001 by log-rank test. Abbreviations: CHD, congenital heart disease; CAA, congenital airway anomaly.

Kaplan–Meier analysis revealed that the cumulative incidence of mortality in children with CHD was significantly higher in those with CAA than in those without (log-rank test, *p* < 0.001) (Fig 1).The differences rapidly became prominent during the 0–3 years after CHD diagnosis, but stabilized thereafter.

Overall, the mortality rate was significantly higher in children with CHD associated with CAA (199 of 1,591; 12.5%) than in those without CAA (2,334 of 38,061; 6.1%) (*p* < 0.001). The increased mortality in children with CHD and CAA remained after stratifying the data by sex (CHD with vs. without CAA: boys, 11.4% vs. 6.5%, *p* < 0.001; girls, 14.1% vs. 5.8%, *p* < 0.001) and age (CHD with vs. without CAA: neonates, 16.9% vs. 8.6%, *p* < 0.001; infants, 8.2% vs. 3.8%, *p* < 0.001; toddlers and preschool children, 6.0% vs. 0.8%, *p* = 0.009). A significant difference in mortality was found in patients with follow-up at 0–3 years (CHD with vs. without CAA: 39.2% vs. 22.2%, *p* < 0.001) and 4–7 years (CHD with vs. without CAA: 2.1% vs. 0.6%, *p* < 0.001). Mortality rates were also higher in children with concomitant CHD and CAA associated with additional nervous system (CHD with vs. without CAA: 21.7% vs. 11.1%, *p* < 0.001), musculoskeletal (CHD with vs. without CAA: 14.9% vs. 7.4%, *p* < 0.001), and genital (CHD with vs. without CAA: 9.3% vs. 3.8%, *p* = 0.044) anomalies. Table 3 summarizes the mortality rates among the CHD study populations with and without CAA by different factors.

**Table 3:** Mortality analysis for children with CHD by CAA status.

	CAA (+)			CAA (-)			
Variables	Total, n	Mortality, n	(Rate, %)	Total, n	Mortality, n	(Rate, %)	*p* ^a^
Overall	1591	199	(12.5)	38061	2334	(6.1)	<0.001
Gender							
Male	946	108	(11.4)	18767	1211	(6.5)	<0.001
Female	645	91	(14.1)	19294	1123	(5.8)	<0.001
Age at CHD diagnosis^a^							
Neonates	800	135	(16.9)	21347	1835	(8.6)	<0.001
Infants	741	61	(8.2)	12056	461	(3.8)	<0.001
Toddlers & Preschools	50	3	(6.0)	4658	38	(0.8)	0.009
Follow-up period, years							
0–3	480	188	(39.2)	10144	2255	(22.2)	<0.001
4–7	478	10	(2.1)	10745	66	(0.6)	0.001
≧8	633	1	(0.2)	17172	13	(0.1)	0.398
Other associated ECA							
Nervous system	157	34	(21.7)	1026	114	(11.1)	<0.001
Musculoskeletal system	202	30	(14.9)	1048	78	(7.4)	0.001
Eyes/ears/face/neck	85	6	(7.1)	390	24	(6.2)	0.756
Cleft lip/palate	110	11	(10.0)	622	56	(9.0)	0.738
Gastrointestinal system	81	16	(19.8)	765	102	(13.3)	0.113
Urinary system	43	6	(14.0)	497	36	(7.2)	0.132
Genital system	86	8	(9.3)	656	25	(3.8)	0.044
Integument	4	1	(25.0)	52	4	(7.7)	0.320
Chromosome	155	33	(21.3)	1112	257	(23.1)	0.613

Abbreviations: CHD, congenital heart disease; CAA, congenital airway anomaly; ECA, extracardiac anomaly.

^a^χ^2^ or Fisher’s exact tests for the comparison about the mortality rate between with and without CAA.

Children with both CHD and CAA had a significantly elevated mortality risk (crude HR, 2.05; 95%CI, 1.77–2.37) than those with CHD without CAA, and this difference remained after adjusting for sex, age at CHD diagnosis, and other comorbidities (adjusted HR [aHR], 1.76; 95%CI, 1.51–2.04). With respect to the stratified analysis of mortality risk in children with CHD and CAA, mortality risk was significantly higher than for CHD without CAA by sex (aHR, 95%CI; boys 1.62, 1.32–1.98; girls, 2.01, 1.61–2.50) and age (aHR, 95%CI; neonates, 1.67, 1.40–2.00; infants, 1.93, 1.47–2.55; toddlers & preschools, 4.77, 1.39–16.44) strata. The overall and stratified results of the Cox regression analyses for mortality risk in patients with CHD and CAA are shown in Table 4.

**Table 4:** Overall and age- and sex-stratified crude and adjusted HRs of mortality risk for patients with CAA and CHD.

		HR (95%CI)			
Strata	CAA	Crude		Adjusted	
Overall	(+)	2.05	(1.77–2.37)	1.76	(1.51–2.04)^a^
	(-)	1.00		1.00	
Gender					
Male	(+)	1.78	(1.46–2.17)	1.62	(1.32–1.98)^b^
	(-)	1.00		1.00	
Female	(+)	2.44	(1.97–3.02)	2.01	(1.61–2.50)^b^
	(-)	1.00		1.00	
Age at CHD diagnosis^d^					
Neonates	(+)	1.98	(1.66–2.35)	1.67	(1.40–2.00)^c^
	(-)	1.00		1.00	
Infants	(+)	2.17	(1.66–2.84)	1.93	(1.47–2.55)^c^
	(-)	1.00		1.00	
Toddlers & Preschools	(+)	6.97	(2.15–22.63)	4.77	(1.39–16.44)^c^
	(-)	1.00		1.00	

Abbreviations: CHD, congenital heart disease; CAA, congenital airway anomaly; HR, hazard ratio; CI, confidence interval.

^a^HRs adjusted for gender, age at CHD diagnosis, and other ECA.

^b^HRs adjusted for age at CHD diagnosis and other ECA

^c^HRs adjusted for gender and other ECA.

^d^Neonate, newborn to ≤ 28 days of life; infant, >28 days but < 12 months; toddler & preschool, aged 1–5 years.

## Discussion

To date, this is the first large-scale nationwide population-based analysis to investigate the factors associated with CAA, and the associated change in mortality risk, in a population of children with CHD. This study of 39,652 children aged 0–5 years with CHD demonstrates that boys, infants, and those with other congenital musculoskeletal anomalies are most likely to have an associated CAA, and that the condition is most likely identified 0–3 years after the diagnosis of CHD. In addition, there is evidence that children with CHD associated with CAA have a significantly elevated mortality risk, with an aHR of 1.76 after a median 7-year follow-up period.

The heart starts to differentiate around the third week of embryonic stage [25], and the airway begins to develop at the fourth week of embryonic life [26]. The temporal and spatial proximity between the development of the cardiovascular and respiratory systems provides them with the simultaneous exposure to intrauterine insults or to the effects of abnormal structural development in each system [5, 18]. CHD could also alter the perfusion to the aortic arches that give rise to the airway structures and be an alternative etiology of CAA [19, 27]. Respiratory symptoms are often presented in infants and children with CHD and generally arise from large left to right shunt, flow obstruction of the systemic ventricle failure, vascular airway compression, and intrinsic pathology [28–30]. CAA associated with CHD may complicate the clinical manifestation and significantly increase mortality and morbidity among these patients [28, 31, 32].

Most studies to date have reported that the prevalence of ECA among children with CHD is 20% to 30%, which is much higher than that in the general population [9, 12, 33, 34]. As for the occurrence of CAA in CHD, previous studies have reported rates of 3% to 4% of children with CHD [5, 9, 12]. However, Pfammatter et al. reported prevalence of airway anomalies at 1.5% in pediatric patients undergoing CHD surgery [16]. In the current study, CAA occurred at a rate of 4.0% among children with CHD, which is comparable with prior literature [5, 9, 12]. The differences in prevalence may be due to differences in study design, with most of the previous reports being hospital-based and relying on limited numbers and subject to possible selection bias. This study uses a nationwide population-based dataset with almost complete coverage of CAA and CHD in the population. Consequently, our data are probably more reliable.

CAA is an often unrecognized problem in children with CHD, and early identification of this congenital respiratory pathology is essential for appropriate management [15, 35]. Because of the intrathoracic contiguity, any pathological alterations in the respiratory or cardiovascular systems may have potential impacts on the other. Moreover, the clinical presentations of CAA and CHD may mimic each other. In children with CAA, although the severity may be comparatively trivial, symptomatic exacerbations and respiratory compromise may become more apparent in patients with comorbid medical condition [36]. For children with CHD, impaired oxygen delivery and altered systemic or pulmonary blood flow can lead to circulatory and respiratory dysfunctions [35]. Additionally, the anatomic cardiovascular compression of the airway may also cause respiratory morbidity in patients with CHD [15, 16]. These features combine to make the timely diagnosis of CAA in children with CHD difficult. The results of this study indicate that children with CHD are most likely to have a CAA if they are boys (OR, 1.43), infants at CHD diagnosis (OR, 5.31), and have other congenital musculoskeletal anomalies (OR, 3.19), and are most likely to be diagnosed during the 0–3 years after the CHD diagnosis (OR, 1.33). To reduce the morbidity and mortality caused by CAA among children with CHD, these results should be used to guide early clinical identification before respiratory compromise can develop.

Though CAA has been noted to occur more frequently in patients with underlying genetic or syndromic disease, such as Trisomy 21, VACTERL, and Klippel-Feil [19, 37–39], few data have been presented that examine the relationship of CAA and other ECA in patients with CHD. The present results show that the probability of CAA was significantly higher in patients with CHD if they had another ECA, such as musculoskeletal (OR, 3.19), eyes/ears/face/neck (OR, 2.72), nervous system (OR, 2.71), chromosomal (OR, 2.51), cleft lip/palate (OR, 2.28), genital (OR, 1.76), or gastrointestinal (OR, 1.59) anomalies. CAA and other ECA may occur concurrently in the same CHD individual, perhaps through some common developmental mechanism. With the genetic basis for CHD becoming clearer and the higher incidence of CAA within this population, it calls for efforts to explore the possible genetic connection of these defects [19]. Further investigations may be warranted to clarify the relationship between CAA and other ECA in patients with CHD if we are to unravel the underlying mechanisms.

The mortality data presented in other studies of children with CHD are conflicting. Jenkins et al. reported that the mortality rate for CHD surgery was 7.3% [40], while Welke et al. reported a rate of 2.9% [41]. In this study, the mortality rate for all children with CHD was 6.4%. The discrepancy in the reported mortality rate may be due to the study populations used, with most previous studies focusing on surgical patients, while the current study investigated all children with CHD. Small sample sizes with limited power may also explain the inconsistent results.

Previous studies have often failed to explore the differences in mortality according to the presence or absence of ECA; in this study, we specifically clarify the CHD mortality associated with the presence of CAA. We have demonstrated that the mortality rate was significantly greater for CHD with CAA (12.5%) than that without CAA (6.1%), and that statistical significance remained when stratifying by sex, age, time after CHD diagnosis (i.e., 0–3 years and 4–7 years), and the presence of congenital nervous, musculoskeletal, and genital anomalies. After adjusting for sex, age, and comorbidities, the added mortality risk of CAA in CHD patients is 1.76 times higher than in those without CAA. Together, these results imply that CAA is an unfavorable prognostic factor in children with CHD. The significantly elevated mortality risk may be due to the long-term interaction of the cardiovascular and respiratory pathologies that lead to increased morbidity [16]. The prior literature suggests that clinicians must be vigilant for respiratory anomalies in patients presenting with progressive respiratory distress [35]. However, our results suggest that children with CHD deserve even greater attention given the increased possibility of co-occurrence with CAA and the significantly elevated mortality risk when CAA does exist concomitantly.

Although boys with CHD (OR, 1.43) were more likely to be diagnosed with CAA, the mortality risk for girls with CHD and CAA (aHR, 2.01) was higher than that of boys (aHR, 1.62). There is no adequate explanation for these sex-based differences among children with both CHD and CAA. Further investigation is needed to understand these differences.

The results of stratified analyses demonstrate that, compared to children with CHD without CAA, children with CHD diagnosed as toddlers and at preschool have the highest mortality risk (aHR, 4.77). The different mortality risk in different age strata may reflect the prolonged and complex pathophysiological interaction between CAA and CHD. The later CHD is diagnosed, the more profound the interaction between the respiratory and cardiovascular systems will be. Additional research is needed to draw meaningful conclusions.

The results of the Kaplan–Meier analysis showed that the cumulative incidence of mortality among children with CHD increased with a steeper slope in the first 0–3 years after CHD diagnosis, and then stabilizes thereafter. This pattern was true of patients with and without CAA, although the magnitude differed between each. In addition, the difference in the cumulative incidence of mortality only became prominent during the first 0–3 years after the CHD diagnosis, implying that this may be a critical period for these children. CAA may be responsible for the differences in cumulative incidence of mortality.

To our knowledge, this is the first large-scale nationwide population-based cohort study to assess the association between CAA and CHD and focusing on the added mortality risk of CAA. The major strengths of the current study are the population-based design, the complete coverage of CAA and CHD in the population (precluding the possibility of loss to follow-up), and the longitudinal follow-up. Nevertheless, this study has some limitations that are worth considering. First, because it is epidemiologic in nature, it cannot establish causative links, only associations. Second, personal information is not documented in the database, including the clinical phenotypes and severities of CAA and CHD, environmental factors, and lifestyle factors. Therefore, an analysis of the possible relationships between these personal characteristics and the mortality risk is not possible. Third, because the study was conducted in Taiwan, the extent to which the findings can be generalized to other population warrants further discussion. Additional large studies, with a longer follow-up period, involving different age strata, and in different countries, are required to make definitive conclusions.

## Conclusion

Children with CHD are most likely to be diagnosed with CAA if they are boys, infants at the time of CHD diagnosis, or have another congenital musculoskeletal anomaly. Further, most children are diagnosed with CAA 0–3 years after the CHD diagnosis. In addition, the overall and the sex- and age-stratified mortality risks are significantly increased in children with CHD and comorbid CAA. We recommend that clinicians be vigilant for CAA during the follow-up of children with CHD. Further studies are warranted to elucidate the potential mechanisms underlying the differences in mortality that we identified in association with CAA.
